# *Mycobacterium chimaera* chorioretinitis preceding central nervous system lesions: a case report and review of the literature

**DOI:** 10.1186/s12886-022-02528-2

**Published:** 2022-07-22

**Authors:** Aaron Veenis, Megan Haghnegahdar, Radwan Ajlan

**Affiliations:** Department of Ophthalmology, University of Kansas School of Medicine, 7400 State Line Rd, Prairie Village, Kansas, KS 66208 USA

**Keywords:** *Mycobacterium chimaera*, Chorioretinitis, Case report

## Abstract

**Background:**

*Mycobacterium chimaera* ocular infection is a rare disease that is linked to bypass devices used during cardiothoracic surgeries. Reported cases in the literature of ocular involvement preceding CNS involvement are based on clinical exam with no neuroimaging. Here we present a case of *M. chimaera* ocular infection with no CNS *M. chimaera* lesions on brain magnetic resonance imaging (MRI).

**Case presentation:**

A 59-year-old female presented with altered mental status and blurred vision in February 2021. Her past medical history was significant for aortic valve replacement and ascending aortic aneurysm repair in 2017 complicated by known *M. chimaera* infection. She had been receiving azithromycin, ethambutol, rifampin, and amikacin as systemic anti-mycobacterium treatment. Her dilated fundus exam showed numerous yellow placoid circular lesions scattered throughout the macula and peripheral retina in both eyes with associated vitritis. Systemic workup, including brain MRI showed no acute infectious lesions. Her infections workup was unremarkable except for a positive toxoplasma IgM, for which she was treated with sulfamethoxazole/trimethoprim. One month later, a head computed tomography showed new numerous scattered round foci of hyperdensity throughout the cerebrum and brainstem thought to be foci of *M. chimaera* infection. Clofazimine was added per culture and sensitivity. MRI brain 1 month later showed mild decrease in conspicuity and number of these intensities while on anti-mycobacterium treatment. Her cognition had improved at that time as well. She was seen in retina clinic 2 months later where her exam showed similar retinal lesions with no associated vitritis or anterior chamber cell in bilateral eyes, suggesting a lack of active infection. Optical coherence tomography macula showed parafoveal cystoid macular edema bilaterally. She was started on steroidal and non-steroidal anti-inflammatory eye drops.

**Conclusions:**

To the best of our knowledge, this is the first case in the literature to report *M. chimaera* chorioretinitis with concomitant negative neuroimaging. Chorioretinal *M. chimaera* lesions should motivate high suspicion of CNS involvement prompting early neurological work up.

## Background

*Mycobacterium chimaera* (*M. chimaera*) is a non-tuberculous mycobacterium that was recognized as part of the mycobacterial *avium* complex in 2004 [1]. Infection often presents as focal respiratory infection or disseminated infection in immunocompromised patients and patients with an underlying respiratory disease [2]. More recently infection from *M. chimaera* has been linked to a bypass device used intraoperatively for various cardiothoracic surgeries [2–5]. These infections often have a delayed presentation of months to years following the cardiothoracic surgery and are oftentimes missed by routine microbiological diagnostic tests [2]. Patients present with non-specific symptoms such as fever, cough, malaise, weight loss, or shortness of breath [2, 6]. Recommended treatment consists of targeted antimicrobial antibiotics, according to susceptibility testing, and surgical excision of the infected cardiovascular hardware [2]. Outcome of these infections has been poor despite the described treatment [2].

Ocular involvement by *M. chimaera* after cardiothoracic surgery is well documented in the literature. The clinical and histopathological findings of five patients in one study demonstrated that all five patients presented with chorioretinal lesions [7]. Few patients presented with anterior uveitis, intermediate uveitis, or optic disc swelling [7]. Multimodal imaging has been used to characterize the morphology of choroidal lesions in patients with progressive ocular *M. chimaera* infection [7, 8]. While enhanced depth imaging optical coherence tomography (EDI-OCT) can detect and monitor active choroidal lesions, indocyanine green angiography and fluorescein angiography were useful in monitoring the total number of lesions and overall disease progression [8].

Lecorche et al. reported *M. chimaera* chorioretinitis 1 month prior to the discovery of central nervous system (CNS) involvement, however no neuroimaging had been completed prior to or at the time of the ocular diagnosis [9]. Three additional cases documented chorioretinal lesions prior to the investigation of encephalitis with neuroimaging [10]. Each of these three cases later developed progressive neurologic symptoms and displayed CNS involvement on neuroimaging confirmed by histopathologic diagnosis [10]. In all previous cases, no neuroimaging was completed when the ocular involvement was discovered and thus, concomitant CNS and ocular involvement cannot be excluded [7, 9, 10]. One case reported systemic disseminated infection which developed CNS and ocular involvement 2 months later [11]. With this case, the timing of CNS and ocular involvement cannot be discerned because they were discovered at the same time [11].

In total, five cases are documented in the literature with *M. chimaera* chorioretinitis prior to the discovery of CNS involvement, but with no neuroimaging available at the time of ocular diagnosis [7, 9, 10]. To the best of our knowledge, we report the first case of *M. chimaera* chorioretinitis with no CNS involvement on brain magnetic resonance imaging (MRI) at the time of presentation.

## Case presentation

**Fig. 1:**
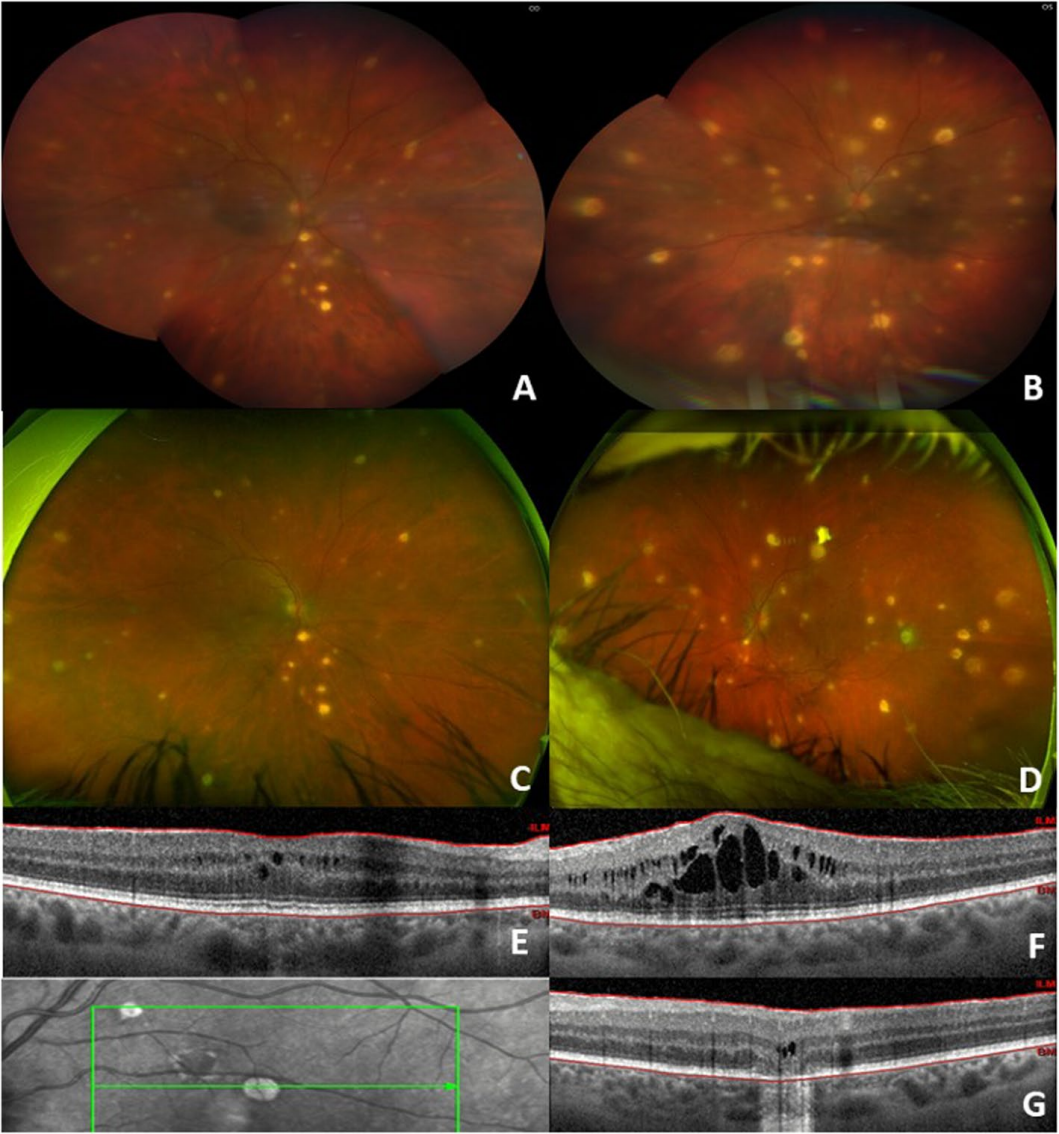
Multimodal imaging of bilateral eyes. Initial ultrawide field fundus photography of the right and left eyes demonstrating numerous yellow placoid circular lesions with associated vitritis (**A**, **B**). Ultrawide field fundus photography of the right and left eyes two months later showing similar appearing lesion with clear media (**C**, **D**). OCT macula of right and left eyes demonstrating cystoid macular edema (**E**, **F**). OCT macula through an inactive lesion with associated raster image showing outer retinal layers atrophy (**G**)

A 59-year-old female presented to the emergency department with altered mental status and blurred vision in February 2021. Her past medical history was significant for aortic valve replacement and ascending aortic aneurysm repair in 2017 complicated by periaortic abscess secondary to *M. chimaera* infection. She underwent repeat aortic valve replacement and ascending aortic aneurysm repair with placement of amikacin beads around the new graft in January 2021. She had been receiving azithromycin, ethambutol, rifampin, and amikacin as systemic anti-mycobacterium treatment. On initial presentation, her dilated fundus exam showed numerous yellow placoid circular lesions scattered throughout the macula and peripheral retina in both eyes with associated vitritis (Fig. 1 A and B). She underwent extensive workup, including brain magnetic resonance imaging (MRI) which showed no acute infectious lesions. Her infectious workup was unremarkable except for a positive toxoplasma IgM, for which she was started on sulfamethoxazole/trimethoprim in addition to her *M. chimaera* treatment regimen. She was then discharged to a skilled nursing facility.

The patient sustained a fall 1 month later and was admitted to the hospital again. Her workup included head computed tomography (CT) which showed new numerous scattered round foci of hyperdensity throughout the cerebrum and brainstem thought to be foci of *M. chimaera* infection. Clofazimine was added per culture and sensitivity from her initial paraaortic abscess. Her cognition markedly improved 1 month later. MRI brain at that time showed mild decrease in conspicuity and number of these intensities while on anti-mycobacterium treatment. She was seen in retina clinic 2 months later where her exam showed similar retinal lesions with no associated vitritis or anterior chamber cell in bilateral eyes, suggesting lack of active infection. Optical coherence tomography (OCT) macula showed parafoveal cystoid macular edema (CME) bilaterally. She was started on non-steroidal anti-inflammatory eye drops and prednisolone acetate eye drops with improvement in CME 4 months later. She is currently on a monthly eye drop taper and will follow up in clinic again in 3 months. The patient demonstrated good adherence to this eyedrop regimen. She tolerated the treatment well without any concerns or complications.

On a follow up phone call with the patient’s partner, she has reflected that she is pleased with her evaluation and treatment at our institution. She feels her vision has improved, which has helped with her daily functions. She is very grateful that our assessment aided in guiding her systemic treatment of the *M. chimaera* infection and hopes the shared knowledge of her case aids the ophthalmology community in addressing this diagnosis in other patients.

## Discussion and conclusions

*M. chimaera* infections have been linked to the use of bypass machines during cardiothoracic surgery. This case describes a patient with disseminated *M. chimaera* infection who was found to have chorioretinitis prior to CNS involvement. Other cases have documented similar presentations but did not obtain neuroimaging at the time of ocular diagnosis. Thus, concomitant ocular and CNS involvement could not be ruled out. This case adds to the literature by demonstrating ocular involvement prior to CNS involvement, confirmed by brain MRI. Our case suggests that ocular involvement of *M. chimaera* infection should point providers to investigate CNS involvement through neuroimaging.

After the *M. chimaera* infection outbreak in 2013 [2–5], authors have identified the heater-cooler units used for bypass during the cardiothoracic procedures as a source of contamination [12–14]. In October of 2015, the US Food and Drug Administration recommended proper sanitation or replacement of these cardiothoracic heater-cooler units by institutions [15]. One study used data from Switzerland to estimate the annual incidence of *M. chimaera* infections to be 156–282 cases per year, across the world [16]. Cases of cardiothoracic surgery related *M. chimaera* infection have been linked to procedures that occurred after the 2015 safety statement, suggesting that this infection remains clinically relevant [17]. In fact, Natanti et al. suggest a possible short-term spike in Italy of *M. chimaera* infections because of the long incubation period and the relatively new knowledge of contaminated heater-cooler units [18].

Diagnosis of *M. chimaera* remains a challenging process for clinicians. Some factors that lead to difficulty in diagnosing *M. chimaera* include the lag time between exposure and disease, the non-specific symptoms experienced, and the specialized test required for diagnosis. There can be a significant lag time between exposure and disease, ranging from months to years with the longest documented lag time being 6 years after cardiothoracic surgery [2, 4, 19, 20]. The symptoms seen with *M. chimaera* infection also have a non-descript presentation with the most common findings including fever, cough, malaise, weight loss, or shortness of breath [2, 6]. Additionally, *M. chimaera* requires mycobacterial specific cultures and remains the most common method for definitive diagnosis [20]. However, *M. chimaera* has a tendency for ocular involvement [7, 21]. This tendency can be a clue pointing toward *M. chimaera* infection and has prompted early ophthalmologic evaluation for these patients.

*M. chimaera* has a preference for ocular involvement [2, 7, 8, 21–25], it is an obligate aerobe and requires high oxygen levels for growth, leading to the thought that the high oxygen tension of the choroid explains the tendency for ocular involvement [21, 25]. The most sighted ocular manifestations are chorioretinal lesions with additional findings including anterior uveitis, intermediate uveitis, optic disc swelling, and macular and retinal neovascularization [2, 7, 8, 21–25]. The choroidal lesions have been characterized extensively with fluorescein angiography, ICG, EDI-OCT, and spectral domain optical coherence tomography (SD-OCT) [7]. Zweifel et al. found that choroidal lesions were hyperfluorescent on fluorescein angiography and hypofluorescent on ICG angiography, correlating with lesions seen on SD-OCT [7]. The hypofluorescence seen on ICG angiography reflects nonperfusion from active or previously active lesions [7, 11]. The hyperfluorescence seen on fluorescein angiography correlated to choroidal thickening and retinal elevation on SD-OCT [7, 25]. These lesions can be further characterized as active or inactive lesions, aiding in the management of these patients. Active lesions on EDI-OCT appear as round lesions with well-defined borders, involving the full choroidal thickness [8]. Inactive lesions on EDI-OCT appear as hyporeflective choroidal areas with poorly defined margins [8]. Characterizing lesions as active or inactive allows clinicians to monitor response to treatment and progression of disease. Böni et al. suggest that clinical examination, fundus photography, and EDI OCT are optimal to evaluate active choroidal lesions, while fluorescein angiography and ICG angiography are optimal to evaluate the total number of choroidal lesions and disease progression [8].

Ocular involvement likely correlates with systemic activity and thus can be used as a marker for systemic activity [7, 8, 22]. Zweifel found that in a cohort of 5 male patients, the number of choroidal lesions correlated with the course of systemic disease [7]. Patients with widespread chorioretinitis died of systemic complications despite targeted antimicrobial therapy, while patients with few choroidal lesions had a more favorable prognosis [7]. Overall, the prognosis of disseminated *M. chimaera* infection is poor with an estimated mortality rate of 50% [2, 6, 9, 26, 27]. Systemic complications of disseminated *M. chimaera* infection can include splenomegaly, cytopenia, osteomyelitis, pneumonitis, hepatitis, nephritis, skin infection, chorioretinitis, cerebral vasculitis, endocarditis, myocarditis, mediastinitis, encephalopathy, and bloodstream infection [2, 10, 11, 19, 21].

In 2020, Hasse et al. published international guidelines for the diagnosis, treatment, and prevention of disseminated *M. chimaera* infection following cardiothoracic surgery [19]. The suggested treatment consists of a prolonged course of azithromycin or clarithromycin, with ethambutol and rifamycin [19]. Amikacin is also recommended and should be continued as tolerated [19]. Antimicrobial susceptibility testing is recommended, when done by an experienced laboratory, and should specifically test for clarithromycin and amikacin susceptibility [19]. Surgical therapy with revision of all cardiovascular prosthetic material is recommended in addition to the aforementioned antimicrobial therapy [19]. Additionally, comprehensive ophthalmologic examination is recommended with level C evidence in patients with suspected or confirmed *M. chimaera* infection and should be repeated on a 2-month basis due to the link between ocular and systemic disease activity [19].

The case presented demonstrates a patient with *M. chimaera* chorioretinitis without concomitant radiographic CNS involvement at the time of diagnosis. The patient later progressed to develop CNS involvement as confirmed by MRI brain. These findings are different from prior reports with concomitant CNS and ocular involvement. This case can clue clinicians into suspected future CNS involvement for patients with evidence of chorioretinitis and prompt monitoring of CNS symptoms and imaging. This case report has limitations as it describes the clinical findings of one patient and thus may not be extrapolated to an entire patient population. Strengths of this case report include the comprehensive review of literature performed and the original reporting of *M. chimaera* chorioretinitis preceding CNS lesions. In conclusion, disseminated *M. chimaera* following cardiothoracic surgery is a deadly infection that presents with a wide range of disease spectrum that clinicians should be aware of. Management requires the collaboration of ophthalmologists, infectious disease specialists, neurologists, and cardiothoracic surgeons. Chorioretinitis from *M. chimaera* with altered mental status should alert providers to CNS involvement. Early involvement of infectious diseases and neurology services is essential to further guide treatment.

## Data Availability

All data generated or analyzed during this study are included in this published article.

## Abbreviations

CNS: Central Nervous System
*M. chimaera*): *Mycobacterium chimaera*
EDI-OCT: Enhanced Depth Imaging Optical Coherence Tomography
MRI: Magnetic Resonance Imaging
CT: Computed Tomography
OCT: Optical Coherence Tomography
CME: Cystoid Macular Edema
ICG: Indocyanine Green
SD-OCT: Spectral Domain Optical Coherence Tomography
